# Isolation of *Beauveria bassiana* from the Chagas Disease Vector *Triatoma infestans* in the Gran Chaco Region of Argentina: Assessment of Gene Expression during Host–Pathogen Interaction

**DOI:** 10.3390/jof6040219

**Published:** 2020-10-12

**Authors:** Linda Vanesa Baldiviezo, Nicolás Pedrini, Marianela Santana, María Constanza Mannino, Lucía Beatriz Nieva, Alberto Gentile, Rubén Marino Cardozo

**Affiliations:** 1 Facultad de Ciencias Naturales, Universidad Nacional de Salta, Av. Bolivia 5150, Salta 4400, Argentina; vanesssabaldi@gmail.com (L.V.B.); lucybnieva@gmail.com (L.B.N.); 2 Instituto de Investigaciones Bioquímicas de La Plata (INIBIOLP), CCT La Plata Consejo Nacional de Investigaciones Científicas y Técnicas (CONICET)—Universidad Nacional de La Plata (UNLP), calles 60 y 120, La Plata 1900, Argentina; marianelasantana@gmail.com (M.S.); constanza.mannino@gmail.com (M.C.M.); 3 Ministerio de Salud Pública de la Provincia de Salta, Salta 4400, Argentina; alberto.gntl@gmail.com

**Keywords:** entomopathogenic fungi, resistant triatomines, biological control, bassianolide, beauvericin, limpet, dual gene expression

## Abstract

A native strain of the entomopathogenic fungus *Beauveria bassiana* (Bb-C001) was isolated from a naturally infected *Triatoma infestans*, Klug (Hemiptera: Reduviidae) adult cadaver in the Gran Chaco region, Salta province, Argentina. The isolate was both phenotypic and molecularly characterized in a context of fungus-insect interaction, by measuring the expression pattern of toxin genes during infection and immune response of *T. infestans*. The commercial strain GHA of *B. bassiana*, which was previously used in field interventions to control these vectors, was used as reference in this study. The phylogenetic trees based on both ribosomal internal transcribed spacer (ITS) and elongation factor 1-alpha (EF1-α) indicated that Bb-C001 fits into a *B. bassiana* cluster, and the sequence-characterized amplified regions (SCAR) showed that Bb-C001 is different from the GHA strain. There were no differences between both strains regarding viability, radial growth, and conidia production, either in the median survival time or insect mortality. However, Bb-C001 showed a higher expression than GHA of the bassianolide synthetase gene (*BbbslS*) during infection, and similar levels of the beauvericin synthetase gene (*BbbeaS*). Immune-related genes of *T. infestans* nymphs (*limpet-2* and *defensin-1, -2,* and *-6*) were later expressed and thus insects failed to stop the infection process. These results showed that *B. bassiana* Bb-C001 is a promised fungal strain to be incorporated in the current biological control programs of *T. infestans* in Salta province, Argentina.

## 1. Introduction

The blood-sucking insect *Triatoma infestans*, Klug (Hemiptera: Reduviidae), the main Chagas disease vector in the Southern Cone of South America, has been the target of continuous control programs to reduce the disease transmission risk. The control strategy used has been the indoor application of pyrethroid insecticides [1]. However, it has been recognized that this strategy has limited efficacy, mainly confined to the Gran Chaco area shared by Argentina, Bolivia, and Paraguay [2]. Moreover, in the last years several foci of pyrethroid-resistant *T. infestans* have been documented in wide regions of Bolivia and Argentina [3,4]. These difficulties make it necessary to search for alternative tools to insect control. The entomopathogenic fungus *Beauveria bassiana* (Ascomycota: Hypocreales) commercial strain GHA has shown to be useful in field interventions, showing promising results in houses infested with pyrethroid-resistant *T. infestans* [5,6].

Fungal pathogenesis depends on many factors that are related to germination capacity, growth rate, and spore yield and production, among other factors [7,8]. But the way in which fungus and host interact is key to knowing the final result of the infection process [9]. *B. bassiana* secretes toxic or immunosuppressive compounds (often referred to as secondary metabolites) during hemocoel invasion, such as the cyclooligomer nonribosomal peptides beauvericin and bassianolide, the diketomorpholine bassiatin, the cyclic peptides beauverolides, the dibenzoquinone oosporein, and the 2-pyridone tenellin [10,11,12]. Even though the genes involved in some of these secondary metabolites biosynthetic pathways have been studied, most of their biological roles remain to be uncovered [11]. In this regard, their expression pattern when the fungus grows within its insect host might help to better understand their role in pathogenesis. Our research group has developed a methodology based on absolute quantification by qPCR to follow it, reporting an induction of the genes encoding for the synthetase enzymes of the secondary metabolites beauvericin (*BbbeaS*), bassianolide (*BbbslS*), and tenellin (*BbtenS*) during the first days of infection, perhaps to be used as virulence factors, and then in moribund insects and/or cadavers to protect them from competitive microorganisms [13]. On the other hand, insects possess both innate cellular and humoral defense strategies to defend from microbe infections. Humoral response comprises the production of many different antimicrobial peptides, including defensins [13]. *T. infestans* contains six genes encoding for defensins, and their expression is regulated at least by a pair of limpet transcription factors [14].

All these properties allow selecting strains with optimal characteristics to achieve effective control results; however, the fungus also need to be host-specific, virulent, and adapted to a regional environment [15]. In this regard, exploration for local isolates is crucial to establish long-term, effective, and sustainable biological control programs. There are only two reports on the isolation of entomopathogenic fungi naturally infecting *T. infestans* in Argentina; i.e., isolates of *B. bassiana* [16] and *Paecilomyces lilacinus* [17]. However, there is abundant information about *T.* infestans-*B. bassiana* interaction [13,18,19,20,21,22], even from a coevolutive perspective [23].

In the present study, we reported the isolation of a native *B. bassiana* strain from a *T. infestans* cadaver. We characterized its phylogeny, growth, virulence, and toxin expression during infection of both pyrethroid-susceptible and pyrethroid-resistant *T. infestans*, and also evaluated the host immune response within the context of a fungus–insect interaction. 

## 2. Materials and Methods 

### 2.1. Fungus Isolation

As part of periodical entomological interventions performed by members of the Ministry of Public Health of Salta Province, Argentina, a cadaver of *T. infestans* adult with apparent signs of mycosis was isolated in August 2011 at the “Misión Aborigen el Cañaveral”, Santa Victoria Este municipality, Rivadavia department, Salta province (22°16′52.78″ S; 62°42′5.60″ W). This place belongs to the Gran Chaco region and is located at the edge of the Pilcomayo River. The cadaver of the infected insect was sterilized with 0.5% sodium hypochlorite and was sowed in a Petri dish with potato dextrose agar (PDA) with chloramphenicol and incubated at 27 °C for 7 d to isolate the fungus. The colony obtained was subcultured several times to obtain a pure culture, which was preliminary identified on the basis of macromorphological aspects [24] as *Beauveria* sp. and conserved at the Mycological Culture Collection of the School of Natural Sciences, National University of Salta, Argentina, under the code Bb-C001. The strain GHA *of B. bassiana* (obtained from Laverlam International, Butte, MT, USA) was used as reference for the entire characterization. In order to regain the optimal virulence parameters, both strains were inoculated on *T. infestans* nymphs and recovered in PDA prior to use [8]. 

### 2.2. Molecular Identification

For the molecular and phylogenetic characterization, fungal mycelia were disrupted using a minibead beater homogenizer (BioSpec, Bartlesville, OK, USA) with glass beads (0.5 mm diameter) as previously described [25]. Genomic DNA was extracted with Tri Reagent^®^ (Molecular Reagent Centre, Cincinnati, OH, USA) and used as template in PCR amplifications to detect sequence-characterized amplified regions (SCAR), ribosomal internal transcribed spacer (ITS), and elongation factor 1-α (EF1-α). Primers used are shown in Table 1. A SCAR marker specific for *B. bassiana* strain GHA was amplified with the thermal profile described by Castrillo et al. [26]. The ITS fragment (~600 bp) and EF1-α amplicon (~1200 bp) were amplified employing the touchdown PCR procedure described by Don et al. [27] and modified by Rehner and Buckley [28]. PCR products were visualized in 1% agarose gels stained with ethidium bromide. A 100-bp ladder standard (Productos Bio-Logicos, Quilmes, Argentina) was also used. Amplicons were ligated and cloned into a pGEM-T Easy vector (Promega, Madison, WI, USA) and transformed into *E. coli* JM109. Ampicillin-resistant colonies were isolated and their plasmid purified (Qiagen, Hilden, Germany). Inserts were sequenced (Macrogen, Seoul, Korea) to confirm their identity and use in phylogenetic analysis. Both ITS and EF1-α sequences corresponding to Bb-C001 were aligned along with sequences from several ARS Collection of Entomopathogenic Fungal Cultures (ARSEF) isolates (including GHA) belonging to the genus *Beauveria* [28]. The sequences from *Cordyceps* cf. *scarabaeicola* (ITS, GenBank AY532058; EF1-α, GenBank AY531967) were selected as out-group. Alignments were generated using ClustalW [29]. Phylogenetic analysis was carried out, and maximum parsimony tree was constructed with the program Mega 6.0 [30]. Gaps were excluded from the analysis. A bootstrap analysis with 1000 replicates was used to infer branch support.

### 2.3. Phenotypic Characterization

Total conidia were collected from PDA cultures at different time periods (see below) and suspended in distilled water containing 0.01% Tween 80. Conidia concentration of each initial suspension was estimated with a Neubauer haemocytometer using serial dilutions up to a factor of 10^−7^. For viability assays, three aliquots (5 µL each) were taken from this mother solution, plated on PDA and incubated at 27 °C. Twenty-four hours later, they were observed under a microscope using a 100× objective. Germination percentage was calculated as the number of germinated conidia/total number of conidia × 100. Both procedures (conidia concentration and viability) were repeated in the different postsowing times (10, 20, and 30 days) to evaluate whether both parameters were time-affected. For radial growth, each strain was inoculated in the center of the 80 mm diameter Petri dish with PDA medium. Photographs were taken every 48 h, and the radial growth was measured using the software Image Tool 3.0, considering four perpendicular diameters. Observations were recorded for 30 days after inoculation. The slope was calculated by lineal regression and expressed as radial growth (mm/h) as described in [8]. For each strain, 12 replicates were done.

### 2.4. Insects

Both pyrethroid-susceptible (Py-S) and pyrethroid-resistant (Py-R) *T. infestans* used in this study come from a well-established colony at the insectary of the School of Natural Sciences, National University of Salta, Argentina. Insects were fed on ketamine-anesthetized rat blood once per each development stage. All animal care and laboratory experimental protocols were carried out following the Regulation of the Institutional Committee for Care and Use of Laboratory Animals and Field Studies (CICUALEC), School of Natural Sciences, National University of Salta, Argentina. The colony was periodically renewed by incorporating first generation, field-collected insects. 

### 2.5. Mortality Bioassays

Fungal suspensions were prepared in sunflower oil at a concentration of 1 × 10^12^ conidia mL^−1^, determined with a hemocytometer. Groups of 15 Py-R insects were used for each fungal strain, which included first (NI) and third stage (NIII) nymphs, and adults (A). Each group was placed in Petri dishes (10 cm diameter) containing 1 mL of each fungal formulation homogenously dispersed with a silicone brush (1.3 × 10^10^ conidia/cm^2^). The insects were allowed to be in contact with it for ten minutes, and then transferred individually to acrylic containers and incubated at 27 °C with a photoperiod of 12:12 h without feeding. As control treatments, Petri dishes sprayed with sunflower oil were used. Mortality was registered daily and dead insects were put in a humid chamber in order to confirm that death was caused by fungal infection. Medium survival time (MST), Maximum survival reached (S%), and Kaplan and Meier survival curves were performed nine days after fungal infection. 

### 2.6. Gene Expression by qRT-PCR

We used the dual gene expression approach for studying the molecular interaction between fungi and insects, according to the protocol described by Lobo et al. [13]. For this, two-week-old fourth-instar nymphs, either Py-S or Py-R, were used one week after a blood meal. Individual insects were immersed for 6 s in aqueous (0.01% Tween 80) conidial suspensions of either 0 (control), 1 × 10^2^, or 1 × 10^4^ conidia mL^−1^. Insects were returned and maintained at the rearing conditions described above. At different time periods (three, six, and nine days after treatment), three live insects were separated, and their total RNA was extracted from both fungus-treated and control insects by employing the Tri Reagent^®^ (Molecular Reagent Centre, Cincinnati, OH, USA) technique, according to manufacturer’s instructions. RNA was quantified by a Nanodrop spectrophotometer (Thermo Scientific, Wilmington, DE, USA), and its integrity was assessed on a 1% (*w*/*v*) agarose gel. Two-step real-time polymerase chain reaction (RT-PCR) was carried out with iScript cDNA Synthesis kit and iQ SYBR Green Supermix (Bio-Rad, Hercules, CA, USA). Amplification was performed on an AriaMx Real-Time PCR (qPCR) Instrument (Agilent Technologies, Santa Clara, CA, USA) employing 40 ng reverse-transcribed total RNA for each sample. Targeted fungal genes were those encoding for enzymes involved in the biosynthesis of some secondary metabolites [13]; i.e., tenellin synthetase (*BbtenS*), beauvericin synthetase (*BbbeaS*) and bassianolide synthetase (*BbbslS*). Insect genes assayed were a limpet transcription factor (*Tilimpet-2*) and three defensin genes (*Tidef-1*, *Tidef-2*, and *Tidef-6*) regulated by limpet [23]. Primers used are listed in Table 1. The following amplification program was used: denaturation at 95 °C for 10 min, followed by 40 cycles with three-segment amplification (30 s at 95 °C for denaturation, 30 s at 55 °C for annealing, and 30 s at 72 °C for DNA chain elongation). In order to confirm that only single products were amplified, a temperature-melting step was then performed. Negative controls were performed by using ‘cDNA’ generated without reverse transcriptase as templates. Reactions containing primer pairs without template were also included as blank controls. The assay was performed in duplicate, and three independent biological replicates were done. To analyze the expression profiles, we applied the NRQ model, consisting of the conversion of quantification cycle values (Cq) into normalized relative quantities (NRQs), the adjustment for differences in PCR efficiency between the amplicons [31], and the normalization with reference genes [32].

### 2.7. Statistical Analyses

Concentration data were analyzed with the nonparametric Mann–Whitney test. In order to assess viability and radial growth speed, time was used as a regression variable. Survival data were compared with the Kaplan Meier test. For gene expression analysis, statistical significance was assessed using a one-way analysis of variance followed by Tukey’s multiple comparison test or an unpaired Student’s *t*-test when it corresponded, depending on the number of experimental groups under analysis. Graph Pad Prism 8.0 software (GraphPad Software, San Diego, CA, USA) was used for statistical analyses.

## 3. Results and Discussion

### 3.1. Molecular and Phenotypic Characterization

**Figure 1:**
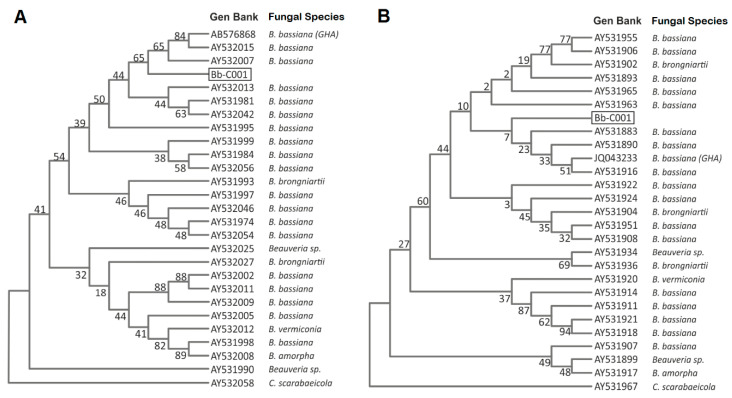
Phylogenetic tree generated by the maximum likelihood analysis of the ribosomal internal transcribed spacer (ITS) (**A**), and elongation factor 1-α (EF1-α) (**B**) sequences of *Beauveria bassiana* Bb-C001 (boxed) and related isolates. The fungal isolates, origin and access numbers to the GenBank are detailed. Numbers on branches are bootstrap support from 1000 replicates.

We reported the isolation of a native fungal strain from a cadaver of an adult specimen of the Chagas disease vector, *T. infestans*. After a first morphological characterization suggesting that the fungus belongs to *Beauveria* sp., we characterized the isolate by molecular tools in order to confirm the identity at species level. The alignment of both ITS and EF1-α fragment sequences showed high similarity (99.5 and 99%, respectively) with *B. bassiana sensu lato* genes (Figure 1A,B) and was named and deposited in local mycological repository (see Material and Methods Section) as Bb-C001. 

**Figure 2:**
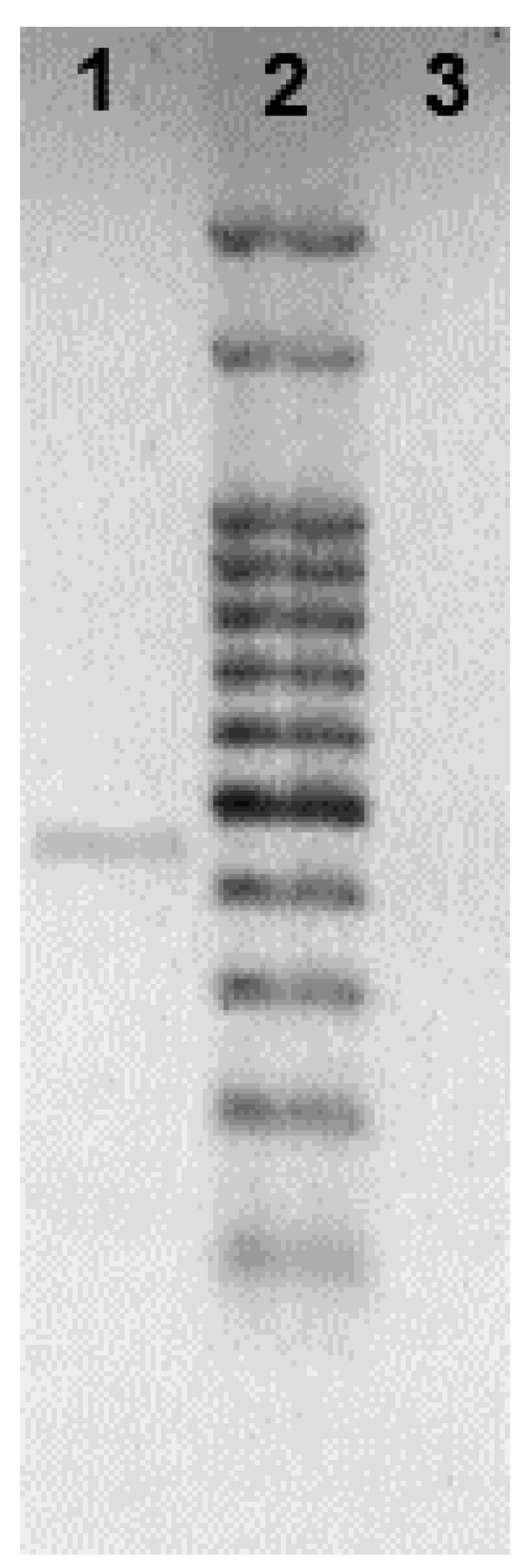
PCR amplification of the sequence-characterized amplified region (SCAR) marker (455 bp) specific for *B. bassiana* strain GHA (lane 1) which is absent in the Bb-C001 isolate (lane 3). Lane 2, molecular weight marker.

Then, as *B. bassiana* strain GHA was previously used in field trials in 2008 and 2009 [5,6] in Salvador Mazza municipality, distant ~130 km from the site of Bb-C001 collection, we assayed a molecular identification using a SCAR marker in order to discard a potential reisolation of naturally dispersed GHA strain from Salvador Mazza to Santa Victoria Este. Sequence-characterized amplified region (SCAR) is a very useful PCR-based technique to differentiate between fungal isolates since it can be developed to be strain-specific. In this sense, Castrillo and coworkers have set a SCAR marker specific for GHA, sensitive enough to differentiate this strain from others *B. bassiana* isolates [26]. Thus, the absence of the SCAR amplicon corresponding to 455 bp in Bb-C001 (Figure 2) confirms that this isolate is different from Bb GHA.

**Figure 3:**
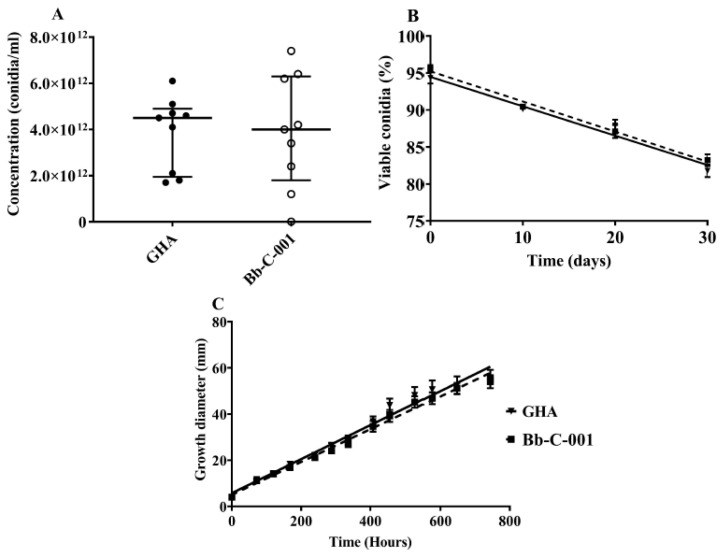
Comparative physiological characteristics of the Bb-C001 and GHA isolates of *Beauveria bassiana*. (**A**) Production of conidia. (**B**) Germination of conidia collected from PDA cultures at different time periods. (**C**) Growth diameter of fungi grown in PDA at different time periods.

Conidia production did not show any statistically significant differences between both strains according to the Mann–Whitney test (*U* = 40.00; *p* = 0.999), with mean values of 4.5 ± 1.6 × 10^12^ conidia mL^−1^ for GHA and 4.0 ± 2.4 × 10^12^ conidia mL^−1^ for Bb-C001 (Figure 3A). Germination percentage did not exhibit statistically significant differences between strains (*p* = 0.59) during the time period studied (0, 10, 20, and 30 days). Regression analysis showed that there is significant loss of viability with time for both stains, GHA (b = −0.4% viability day-1; *p* = 0.02) and Bb-C001 (b = −0.4% viability day-1; *p* = 0.01) (Figure 3B). Radial growth did not exhibit statistically significant differences between the assessed strains (*p* = 0.48), showing an increment in diameter with time. Values obtained were 0.074 ± 0.003 mm h^−1^ (GHA) and 0.071 ± 0.002 mm h^−1^ (Bb-C001) (Figure 3C). 

### 3.2. Fungal Virulence Assessment

**Figure 4:**
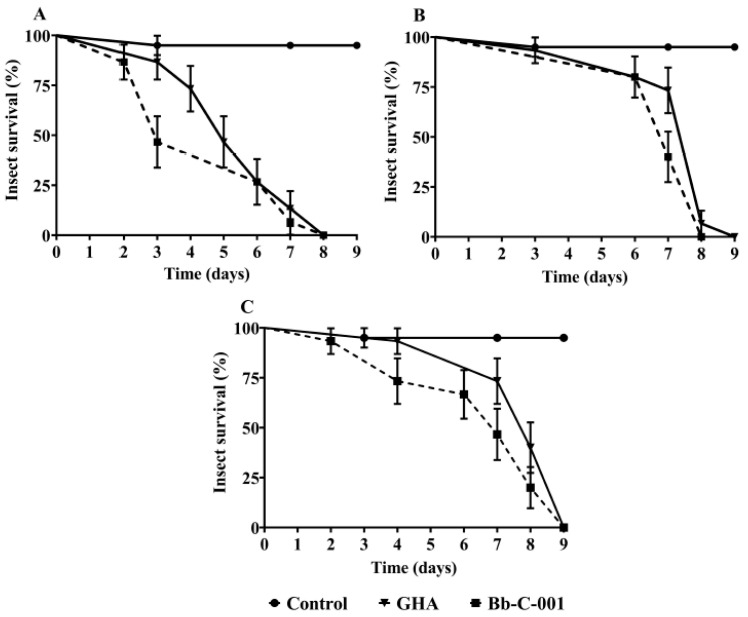
Survival curves of *Triatoma infestans* exposed to both Bb-C001 and GHA isolates of *Beauveria bassiana*, and controls (healthy insects). (**A**) First instar nymphs. (**B**) Third instar nymphs. (**C**) Adults.

Nine days after infection with fungal isolates, no survivors were observed in all *T. infestans* development stages studied (Figure 4). Mean lethal time (MLT) observed for Bb-C001 were 4.6 ± 0.5 days (first instar nymph), 7.2 ± 0.2 days (third instar nymph), and 6.7 ± 0.5 days (adults), whereas MLT obtained for GHA was 5.5 ± 0.4 days (first instar nymph), 7.4 ± 0.4 days (third instar nymph), and 7.9 ± 0.3 (adults). In relation to the survival rate, no statistically significant differences were observed according to the Kaplan and Meier tests between both strains. The isolation and use of native strains of entomopathogenic fungi generate a greater specificity in the infection process, since they are better adapted to the local natural conditions and their host [22,33,34,35].

### 3.3. Fungal Toxin Expression 

**Figure 5:**
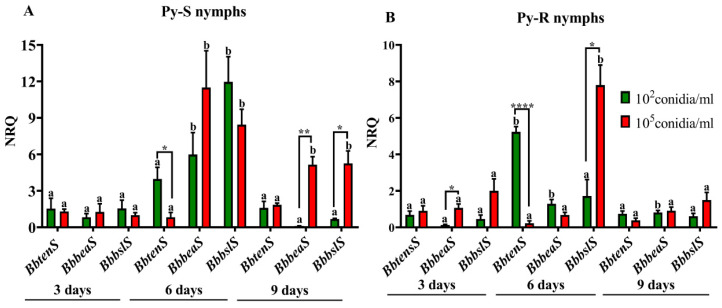
Normalized relative quantities (NRQ) of *Beauveria bassiana* transcripts encoding tenellin (*BbtenS*), beauvericin (*BbbeaS*) and bassianolide (*BbbslS*) synthetases into fourth instar nymphs of pyrethroid-susceptible (**A**) and pyrethroid-resistant (**B**) *Triatoma infestans* at different time periods after insect immersion in conidial suspensions. Values are means of three replicates ± SEM. Different letters indicate significant differences for a single gene through time. Asterisks indicate significant differences in gene expression at each time point. * *p* < 0.05; ** *p* ˂ 0.005; **** *p* ˂ 0.00005.

The expression pattern of Bb-C001 genes encoding for nonribosomal peptides was dependent on the type of nymphs inoculated (Py-S or Py-R), the conidial concentration used (1 × 10^2^ or 1 × 10^5^ conidia mL^−1^), and the time period assayed (three, six, or nine days after fungal inoculation). Even at low values, all three genes (*BbbslS*, *BbbeaS*, and *BbtenS*) were detected inside both Py-S and Py-R nymphs three days after inoculation. In Py-S insects treated with 1 × 10^2^ conidia mL^−1^, the *BbbslS* gene peaked at day six, showing significant higher values than three and nine days post inoculation. *BbbeaS* also peaked at day six, but with lower values than *BbbslS*. *BbtenS* showed the lowest values at the three times assayed (Figure 4A). In Py-S treated with 1 × 10^5^ conidia mL^−1^, *BbbslS* and *BbbeaS* also exhibited the highest values at day six post inoculation, with not significantly different values as on day nine after inoculation (Figure 5A). With respect to Py-R, also higher values were detected at day six post inoculation, *BbtenS* peaked with 1 × 10^2^ conidia mL^−1^, and *BbbslS* with 1 × 10^5^ conidia mL^−1^ (Figure 5B). Comparing both Py-S and Py-R, *BbbslS* and *BbbeaS* genes were higher expressed inside Py-S than in Py-R nymphs at day six post immersion in 1 × 10^2^ conidia mL^−1^ (*BbbslS*, *p* < 0.001; *BbbeaS*, *p* < 0.01) and in 1 × 10^5^ conidia mL^−1^ (*BbbeaS*, *p* < 0.001), and also at day nine post inoculation with 1 × 10^5^ conidia mL^−1^ (*BbbslS*, *p* < 0.05; *BbbeaS*, *p* < 0.01).

The same three genes were measured in GHA strain during infection of the same host [13]. Although both strains exhibited a peak of expression at day six post inoculation, only *BbbeaS* was highly expressed in by both *B. bassiana* strains into Py-S insects. The accompanying gene with high expression was different, namely *BbtenS* in GHA [13], and *BbbslS* in Bb-C001 (this study). Although this result is bounded to a small piece of research, it might be possible that the expression of the plethora of secondary metabolites described in entomopathogenic fungi is being isolate-dependent but also is likely to depend upon the exposure level (i.e., the inoculum) to the pathogen and the physiological state or host condition (e.g., the pyrethroid-related behavior). In this regard, Pedrini et al. [5] have previously studied the susceptibility of both Py-S and Py-R insects to *B. bassiana*. They found no differences in fungal virulence towards different nymphal stages from both insect populations [5]. The same study demonstrates that although a cuticle thickening and higher surface hydrocarbon content of Py-R bugs (compared with Py-S insects) might be related to a reduced penetration of the pyrethroid, and thus contribute to decrease the effective dose of insecticide, these differences do not seem to affect the fungal contact and penetration through the cuticle [5]. However, the current study found some differences in fungal toxin production either inside Py-S or Py-R insects. We can speculate that these different barriers might be responsible for differences of the fungal inoculum starting the infection, and thus, ultimately, provoke differential expression of secondary metabolites within the insect body invasion process.

### 3.4. Insect Immune Response 

**Figure 6:**
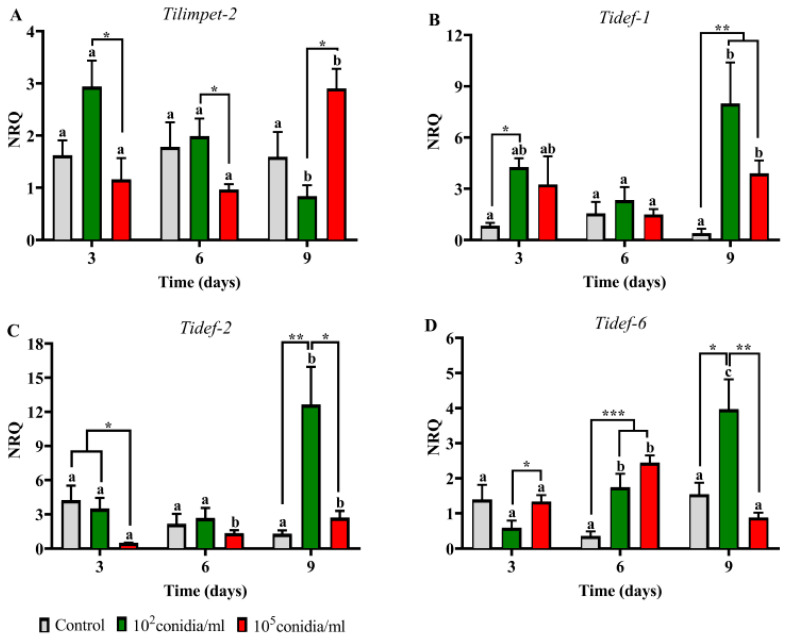
Expression pattern of limpet (**A**) and defensin (**B**–**D**) genes in *Beauveria bassiana*-infected pyrethroid-susceptible nymphs of *Triatoma infestans*. Normalized relative quantities (NRQ) are shown at different time periods after insect immersion in conidial suspensions. Values are means of three replicates ± SEM. For each gene, different letters indicate significant differences for each treatment through time. Asterisks indicate significant differences in gene expression at each time point. * *p* < 0.05; ** *p* ˂ 0.005; *** *p* ˂ 0.0005.

The immune-related gene expression was also different between Py-S and Py-R insects, and the inductions were observed mostly at day nine post inoculation. Comparing with controls, Py-S nymphs treated with 1 × 10^2^ conidia mL^−1^ showed induction in the all three defensin genes at day nine post inoculation, and *Tidef-6* was induced also at day six post inoculation. At the higher dose assayed (1 × 10^5^ conidia mL^−1^), the genes induced were *Tidef-1* at day nine and *Tidef-6* at day six post inoculation (Figure 6). *Tilimpet-2* was not induced either with time period or dose used. 

**Figure 7:**
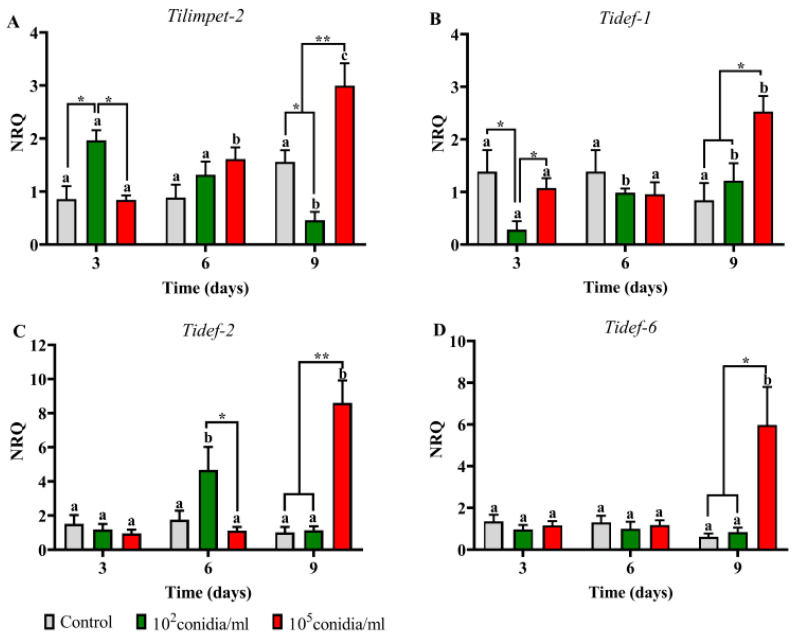
Expression pattern of limpet (**A**) and defensin (**B**–**D**) genes in *Beauveria bassiana*-infected pyrethroid-resistant nymphs of *Triatoma infestans*. Normalized relative quantities (NRQ) are shown at different time periods after insect immersion in conidial suspensions. Values are means of three replicates ± SEM. For each gene, different letters indicate significant differences for each treatment through time. Asterisks indicate significant differences in gene expression at each time point. * *p* < 0.05; ** *p* ˂ 0.005.

In Py-R insects, *Tilimpet-2* was induced at day three after treatment with 1 × 10^2^ conidia mL^−1^ and at day nine with 1 × 10^5^ conidia mL^−1^, as same as the three defensin genes. *Tidef-2* was also induced at day six after treatment with 1 × 10^2^ conidia mL^−1^ (Figure 7). Comparing the immune response in both Py-S and Py-R at day nine post inoculation with 1 × 10^2^ conidia mL^−1^, *Tidef-1*, *-2* and *-6* were more expressed in Py-S than in Py-R nymphs (*p* < 0.001, *p* < 0.001, *p* < 0.05, respectively). On the contrary, in insects inoculated with 1 × 10^5^ conidia mL^−1^ at same time period, Py-R showed higher expression in *Tidef-2* (*p* < 0.001) and *Tidef-6* (*p* < 0.001).

Two variants of the limpet transcription factor were previously characterized in *T. infestans*, and their function have been linked with the humoral innate immune response [14]. As *Tilimpet-2* showed to be more involved than *Tilimpet-1* in defensin regulation, and the most affected defensin genes after limpet silencing were *Tidef-1*, *Tidef-2* and *Tidef-6* [14], we selected these genes to follow the host response in the current study. Although we found similar results as those in the previous work, the role of *Tilimpet-2* in this process (i.e., peaking before defensing genes) was observed only in Py-R insects (day three and day six for limpet and defensins, respectively), but no differences in its expression were detected in Py-S insects. As the defensin genes were more expressed in Py-S nymphs, it might be possible that the induction of *Tilimpet-2* has taken place before day three and thus it has not been detected by this study design.

## 4. Conclusions

In the current study, we compared several aspects of the interaction of two *B. bassiana* strains (GHA and Bb-C001) with the Chagas disease vector *T. infestans*. Both fungal strains did not show any differences on physiological parameters evaluated; i.e., conidial yield, viability, and radial growth, the same as virulence against nymphs and adults. However, the expression of genes encoding nonribosomal peptides was quite different, being the bassianolide syntethase gene the most expressed in Bb-C001, and beauvericin and tenellin synthesase genes the most expressed ones in GHA [13], in all cases peaking at day six post infection. The immune response by measuring the expression of antimicrobial peptides was late and peaked at day nine post inoculation, when the infection process seems to be irreversible. However, differences in the expression of these immune-related genes were observed between pyrethroid-susceptible and pyrethoid-resistant insects. In summary, the infection with both strains develops with similar virulence but the expression of antimicrobial peptides inside insects is different between strains. This finding is an important conclusion from this work, because it confirms that once the fungus reaches the hemocoel, the insect has very little chance to survive the fungal infection despite the activation of the immune response as a last-ditch effort to overcome the fungus. Thus, the main battle in this fungus-insect interaction is to try enter the host by any of the different routes available [36], which determines either a successful infection and the death of the host or an effective defense by the host. Overall, we conclude that the strain coming from of the Gran Chaco region (Bb-C001) is a promissory candidate for the development of a fungal formulation to control both pyrethroid-susceptible and pyrethroid-resistant *T. infestans*. Further studies are underway to evaluate massive production and formulation methods for this strain.

## Figures and Tables

**Table 1:** Oligonucleotides used in this study.

Name	Forward (5′-3′)	Reverse (5′-3′)	Amplicon (bp)	Target	Assay
OPA14F/OPA14R	TCTGTGCTGGCCCTTATCG	TCTGTGCTGGGTACTGACGTG	455	Fungus	SCAR
BbITS4/BbITS5	TCCTCCGCTTATTGATATGC	GGAAGTAAAAGTCGTAACAAGG	~600	Fungus	ITS
BbEF1T/Bb1567R	ATGGGTAAGGARGACAAGAC	ACHGTRCCRATACCACCSATCTT	~1200	Fungus	EF1-α
qBbtenS	ACTGTCCGCATTGGCAGCTAAG	TGTCCTTTGGTGGTGGTGATGG	113	Fungus	qRT-PCR
qBbbeaS	GTTCTTCCTCCGCATTCCGTTC	TAGAGCGCAACGTCTTTCGGTC	97	Fungus	qRT-PCR
qBbbslS	CAATCGACTGAGACGCCATTCC	TTTGACCTGCGAATCCATACGG	156	Fungus	qRT-PCR
qTi18S	GGCGGGGGCATTCGTATTG	ATCGCTGGCTGGCATCGTTTAT	123	Insect	qRT-PCR
qTiEF1	AAAGTGCGACCGTCGTACAGG	TCACGAACGGCAAAGCGA	100	Insect	qRT-PCR
qTiLp2	GCATTTCTGCCAAGAAGAGG	ATGGAATCAAAGCTGGCCTA	110	Insect	qRT-PCR
qTiDef1	TGACTTAGCCCAGCAACCAT	GCACAGGCTGCATGATTAGG	150	Insect	qRT-PCR
qTiDef2	CTTCTTAGTAGCCGCCCTCG	GGTGCCACCATCGTATCCAT	210	Insect	qRT-PCR
qTiDef6	TGAAGTGTGCACTCTCTTTGGT	GGCTCCCATAGGGCTTCATC	106	Insect	qRT-PCR
